# Supplementary material for: Medial orbitofrontal inactivation does not affect economic choice
Source: eLife. 2018 Oct 3;7:e38963. doi: 10.7554/eLife.38963 (PMC6170187; doi:10.7554/eLife.38963)
Supplement: Supplementary file 1. [file elife-38963-supp1.docx]

# Indifference Point

| **Factor** | **Degrees freedom** | **F - score** | **p - value** |
| --- | --- | --- | --- |
| Fiber | 1,94 | 0.01 | 0.93 |
| Laser | 1,94 | 1.71 | 0.19 |
| Virus | 1,94 | 1.28 | 0.26 |
| Fiber*Virus | 1,94 | 1.11 | 0.30 |
| Fiber*Laser | 1,94 | 2.93 | 0.09 |
| Laser*Virus | 1,94 | 0.02 | 0.89 |
| Fiber*Laser*Virus | 1,94 | 3.24e-4 | 0.99 |

**Inverse Slope (Sigma)**

| **Factor** | **Degrees freedom** | **F - score** | **p - value** |
| --- | --- | --- | --- |
| Fiber | 1,94 | 0.32 | 0.57 |
| Laser | 1,94 | 0.61 | 0.44 |
| Virus | 1,94 | 0.25 | 0.62 |
| Fiber*Virus | 1,94 | 0.80 | 0.37 |
| Fiber*Laser | 1,94 | 0.21 | 0.65 |
| Laser*Virus | 1,94 | 0.27 | 0.60 |
| Fiber*Laser*Virus | 1,94 | 0.17 | 0.68 |

**Supplementary File 1. Three-way ANOVA Results Comparing Medial OFC Inactivation with Behavioral Measures from a Virus Control Group.**

Results of a three-way ANOVA with factors Fiber (blocked/patent), Laser (on/off) and Virus (halo/eYFP) for the data shown in **Figure 3-figure supplemental 1 and Figure 4-figure supplemental 1**. Results are shown for both the indifference point (top) and inverse slope (bottom) measures. Effects (main and interactions) are listed by row with the corresponding degrees of freedom, F-scores and p-values.
