# Supplementary material for: Medial orbitofrontal inactivation does not affect economic choice
Source: eLife. 2018 Oct 3;7:e38963. doi: 10.7554/eLife.38963 (PMC6170187; doi:10.7554/eLife.38963)
Supplement: Supplementary file 2. [file elife-38963-supp2.docx]

# Indifference Point

| **Factor** | **Degrees freedom** | **F - score** | **p - value** |
| --- | --- | --- | --- |
| Fiber | 1,147 | 0.08 | 0.77 |
| Laser | 1,147 | 0.33 | 0.56 |
| Fiber*Virus | 1,147 | 1.36 | 0.24 |

**Inverse Slope (Sigma)**

| **Factor** | **Degrees freedom** | **F - score** | **p - value** |
| --- | --- | --- | --- |
| Fiber | 1,147 | 2.29 | 0.13 |
| Laser | 1,147 | 4.27 | 0.04 |
| Fiber*Virus | 1,147 | 1.73 | 0.19 |

**Supplementary File 2. Results of Two-Way ANOVA with Factors Laser and Fiber Performed on a Subset of Trials Taken from the Beginning of each Session.**

Results of a two-way ANOVA with factors Fiber (blocked/patent) and Laser (on/off) performed using the indifference point (top) and inverse slope (bottom) measures determined using data taken from the beginning of each session (first 4 trials of each offer within each session). Effects (main and interactions) are listed by row with the corresponding degrees of freedom, F-scores and p-values.
