# Supplementary material for: Medial orbitofrontal inactivation does not affect economic choice
Source: eLife. 2018 Oct 3;7:e38963. doi: 10.7554/eLife.38963 (PMC6170187; doi:10.7554/eLife.38963)
Supplement: Supplementary file 3. [file elife-38963-supp3.docx]

| **Blocked** | | | | | |  | **Patent** | | | | | |
| --- | --- | --- | --- | --- | --- | --- | --- | --- | --- | --- | --- | --- |
| **A vs C** | | **A vs B** | | **B vs C** | |  | **A vs C** | | **A vs B** | | **B vs C** | |
| **Off** | **On** | **Off** | **On** | **Off** | **On** |  | **Off** | **On** | **Off** | **On** | **Off** | **On** |
| 1.37 | 1.12 | 1.13 | 1.13 | 1.07 | 1.01 |  | 1.53 | 1.33 | 1.51 | 1.50 | 0.96 | 1.21 |
| 1.33 | 1.75 | 1.49 | 1.55 | 0.96 | 0.68 |  | 1.65 | 1.77 | 0.95 | 1.04 | 1.01 | 1.03 |
| 1.25 | 1.42 | 1.66 | 1.25 | 1.08 | 0.96 |  | 1.41 | 1.58 | 1.07 | 0.92 | 1.49 | 1.24 |
| 1.57 | 1.69 | 1.39 | 1.34 | 0.83 | 0.84 |  | 1.66 | 1.65 | 1.49 | 1.46 | 0.77 | 0.80 |
| 1.79 | 1.96 | 1.43 | 1.67 | 1.05 | 1.06 |  | 1.51 | 1.88 | 1.08 | 1.42 | 1.09 | 1.13 |
| 1.56 | 1.60 | 1.30 | 1.50 | 1.22 | 1.52 |  | 1.60 | 1.82 | 1.51 | 1.86 | 0.83 | 0.99 |
| 1.22 | 2.21 | 0.94 | 1.09 | 1.59 | 1.73 |  | 1.87 | 1.88 | 1.18 | 1.14 | 1.66 | 1.22 |
| 1.90 | 1.51 | 1.56 | 1.46 | 1.03 | 1.03 |  | 1.86 | 1.88 | 2.00 | 1.27 | 1.30 | 1.04 |
| **1.90** | **1.96** | **1.68** | **1.45** | **1.74** | **1.40** |  | **2.31** | **1.94** | **1.57** | **1.55** | **1.37** | **1.51** |
| 2.18 | 2.72 | 1.76 | 2.26 | 1.15 | 1.50 |  | 1.96 | 1.89 | 1.82 | 1.49 | 0.90 | 0.99 |
| 2.40 | 2.56 | 2.00 | 2.15 | 1.23 | 1.16 |  | 1.81 | 2.37 | 1.39 | 1.80 | 1.06 | 1.13 |
| 2.42 | 2.82 | 1.62 | 2.54 | 0.79 | 0.84 |  | 2.61 | 3.00 | 1.77 | 2.48 | 1.28 | 1.14 |
| 2.55 | 2.95 | 1.90 | 2.27 | 1.17 | 1.13 |  | 3.10 | 3.05 | 2.36 | 1.88 | 1.08 | 0.91 |
| 3.98 | 4.10 | 2.29 | 1.58 | 2.73 | 2.28 |  | 3.14 | 3.20 | 1.91 | 2.23 | 1.97 | 1.89 |
| 5.57 | 5.75 | 2.47 | 2.77 | 2.59 | 2.52 |  | 5.78 | 3.87 | 2.70 | 2.15 | 2.39 | 2.38 |
| Transitive session sets with IPs out of the IP < 6 range (sessions marked in red) | | | | | | | | | | | | |
| 4.27 | 3.83 | 2.99 | 3.56 | 1.75 | 1.53 |  | 7.22 | 5.04 | 2.23 | 2.47 | 1.79 | 1.79 |
| 5.17 | 4.83 | 0.84 | 0.84 | 2.52 | 1.92 |  | 6.14 | 5.36 | 1.01 | 0.83 | 2.64 | 2.57 |

**Supplementary File 3. Indifference Points for Sets of Transitivity Measures across Three Pellet- Types.**

Indifference points are shown for each experimental unit of transitivity (each row, n = 15, corresponds to data in **Figure 5B**) which comprised of 6 sessions in which each rats received each of the three possible pairings of three pellets for both the blocked and patent fiber conditions (example of a set of sessions is shown in **Figure 5A** which corresponds to the row in bold). Each row shows data for the full design of one experimental unit – a single rat’s behavior on the choice task for each pairing of three pellets A,B, and C (A the most preferred and C the least, as determined by the IPs) for consecutive sessions with the blocked (left 6 columns) and patent (right 6 columns) fiber conditions. IPs for Laser-off (white) and laser-on (grey) trials from each session are shown for each pellet-pair (A vs C, A vs B, B vs C) under each fiber condition for a total of 12 IPs comprising a single experimental unit of transitivity. The last two rows are transitivity sets which were not included in the analysis due to estimated IPs being outside the range of offers given.
